# *Pistacia terebinthus* Resin as Yeast Immobilization Support for Alcoholic Fermentation

**DOI:** 10.3390/foods8040127

**Published:** 2019-04-17

**Authors:** Michalis Kallis, Konstantinos Sideris, Nikolaos Kopsahelis, Loulouda Bosnea, Yiannis Kourkoutas, Antonia Terpou, Maria Kanellaki

**Affiliations:** 1 Food Biotechnology Group, Department of Chemistry, University of Patras, GR-26500 Patras, Greece; m.kallis@me.com (M.K.); konsideris83@gmail.com (K.S.); Kopsahelis@upatras.gr (N.K.); louloudabosnea@gmail.com (L.B.); aterpou@upatras.gr (A.T.); 2 Department of Food Science and Technology, Ionian University, GR-28100 Argostoli, Kefalonia, Greece; 3 Hellenic Agricultural Organization DEMETER, Dairy Research Institute, GR-45221 Ioannina, Katsikas, Greece; 4 Laboratory of Applied Microbiology & Biotechnology, Department of Molecular Biology & Genetics, Democritus University of Thrace, GR-68100 Alexandroupolis, Greece; ikourkou@mbg.duth.gr

**Keywords:** *Pistacia terebinthus* resin, immobilization, *Saccharomyces cerevisiae* AXAZ-1, alcoholic fermentation, phenolic content, volatiles, low-alcohol beverages

## Abstract

A natural resin retrieved from *Pistacia terebinthus* tree was evaluated as an immobilization carrier of *Saccharomyces cerevisiae* AXAZ-1 cells targeting successive fermentation batches of sugar synthetic mediums. Fermentation times below 54 h were recorded at temperatures 28–14 °C. In total, 147 compounds were detected using gas chromatography-mass spectrometry (GC-MS) analysis, including alcohols, esters, ketones, aldehydes, acids, and terpenes. Principal component analysis indicated that the state of cells (free/immobilized) and the fermentation temperature primarily affected terpenes’ composition. Importantly, no spoilage of the fermented beverages was noted during 90 days of storage at room temperature, most likely due to the high content of extracted terpenoids and phenols (up to 579.01 mg L^−1^ and 171.8 mg gallic acid equivalent L^−1^, respectively). Likewise, the developed novel biocatalyst (yeast cells immobilized within *Pistacia terebinthus* resin) was suitable for the production of low alcohol beverages with an enhanced aromatic profile. The obtained results revealed that the proposed bioprocess shows great commercialization potential in the new fast-growing low-alcohol beverages sector.

## 1. Introduction

*Pistacia terebinthus* L. (*Anacardiaceae* family) is a resin-producing tree mainly distributed in the Mediterranean region and Asia, as it preferably grows on dry rock slopes, hill sides, and pine forests [1,2]. Specifically, flowering season of the tree is reported to occur from March to May while its geographical distribution is mainly reported in Greece, Cyprus, Western Turkey, Morocco, Portugal, and the Canary Islands [3]. Different parts of the *P. terebinthus* tree have been reported to possess many biological activities as they have been widely used in phytopharmacy from ancient times [4,5,6]. 

Nowadays, pharmaceutical attention has been drawn on constituents like phenolic compounds, terpenoids, monoterpenes, flavonoids, fatty acids, alkaloids, saponins, and sterols retrieved from *P. terebinthus* for medicinal applications [3,6,7]. The crude extracts, essential oils, and some triterpenoid constituents of *P. terebinthus* might be exploited for therapy of a multitude of inflammatory diseases such as treatments of eczema, paralysis, diarrhea, throat infections, renal stones, jaundice, asthma, and stomachaches, and as an astringent, anti-inflammatory, antipyretic, antimicrobial, antiviral, pectoral, and stimulant [7,8,9,10]. Furthermore, the resin retrieved from *P. terebinthus* possess many applications as it has been traditionally used as chewing gum and a food additive, against lip dryness, certain stomach diseases, and as an antiseptic for the respiratory system [11,12]. Recent studies have revealed the use of this resin as an antimicrobial agent and food preservative as well as a bacterial immobilization carrier for the production of novel functional food products with enhanced aromatic characteristics [2,12,13].

Cell immobilization in alcoholic fermentation is a rapidly expanding research area mostly due to its attractive technical and economic advantages compared to the conventional free cell systems [14,15]. Likewise, many recent studies have reported the advantages of cell immobilization in food production, such as enhanced fermentation productivity [16], enhanced cell stability and viability [17], ability for cell recycling [18], applications of continuous configuration [19], as well as improvement of aromatic characteristics and quality [20]. Although many immobilization supports have been proposed in fermentation systems, inorganic supports are considered inconvenient for food production, while the use of most organic supports, such as alginates, hardly offered an advantageous alternative, thus in many cases it was abandoned [21]. On the other hand, various natural supports, such as fruit pieces [22,23], grape skins [24,25], resins [20], brewers’ spent grains, and delignified cellulosic residues [26] have been investigated and successfully applied as immobilization supports of yeasts and lactic acid bacteria (LAB) for alcoholic and/or milk fermentation. 

The production of alcoholic beverages by yeast fermentation represents one of the most frequently used global biotechnologies [27]. Consequently, *Pistachia terebinthus* resin was applied as a natural immobilization support of the yeast strain AXAZ-1, aiming at successive alcoholic fermentation batches of glucose, sucrose, and maltose at various temperatures. Subsequently, the extracted terpene compounds and phenolic content of the fermented alcoholic liquids were studied and finally the effect of *P. terebintus* resin on the preservation of the fermented alcoholic beverages was monitored. 

## 2. Materials and Methods

### 2.1. Yeast Strain 

The alcohol-resistant and psychrotolerant yeast strain *Saccharomyces cerevisiae* AXAZ-1, previously isolated from the agricultural area of North Achaia (Greece) [28] and sequenced by Kopsahelis et al. (2009) [29], was selected for the fermentation process. The microbial incubation was performed at 30 °C under aerobic conditions (500 cm^3^/min, 0.007 bar), in a synthetic medium containing: 40 g L^−1^ of glucose, 4 g L^−1^ yeast extract, 1 g L^−1^ (NH_4_)_2_SO_4_, 1 g L^−1^ KH_2_PO_4_, and 5 g L^−1^ MgSO_4_ (Merck, Darmstadt, Germany). The produced wet biomass was harvested by centrifugation at 4225× *g* for 10 min (Sigma 3K12, Bioblock Scientific, Lezennes, France) and then stored at 5 °C. All media were autoclaved at 120 °C and at 1–1.5 atm for 15 min, prior to use.

### 2.2. Yeast Cells Immobilization within Pistacia Terebinthus Resin

*Pistacia terebinthus* resin was obtained from Aphrodite Delights Ltd. (Pafos, Cyprus). The immobilized biocatalyst was prepared by mixing 20 g of wet biomass of AXAZ-1 cells and 250 g of crude or melted pieces of *Pistacia terebinthus* resin per L of synthetic medium. Specifically, the priory harvested wet biomass of *S. cerevisiae* (Section 2.1) was added in each flask containing the synthetic medium and pieces of *P. terebinthus* resin and the mixtures were incubated at 28 °C for 24 h to allow cell immobilization by natural adsorption and entrapment [2,13]. The immobilization process was carried out by addition of *P. terebinthus* resin and yeast biomass into a sterile synthetic medium consisting of 120 g L^−1^ of carbohydrates (glucose, sucrose, or maltose depending on the fermentation medium), 4 g L^−1^ of yeast extract, 1 g L^−1^ (NH_4_)_2_SO_4_, 1 g L^−1^ KH_2_PO_4_, and 5 g L^−1^ MgSO_4_ (Merck, Darmstadt, Germany). In order to obtain the optimum conditions for production of the most effective immobilized biocatalyst, the following combinations of yeast cell biomass and *P. terebinthus* resin were tested in 1 L of synthetic medium respectively: 10 and 125; 20 and 250; 40 and 500, wet weight yeast cell biomass (g) mixed with *P. terebinthus* resin (g) accordingly. The fermented liquid was decanted, and the immobilized biocatalyst was washed twice with the sterile synthetic medium for the removal of free cells [2,30]. 

### 2.3. Monitor of Yeast Cells’ Immobilization 

For determination of the immobilized yeasts cells, each immobilized biocatalyst along with the exact same weight of *P. terebinthus* resin were frozen accordingly at −42 °C with a cooling rate of 3 °C/min in a controlled rate freezer (BioCool, FTS Systems, NY, USA). The frozen samples were freeze dried under vacuum (5 × 10^−3^ bar) for 48 h in a Freeze−Drying System (Freezone 4.5, Labconco, Kansas City, MO USA) [2]. The condenser temperature was −45 ± 1 °C. The amount of freeze−dried immobilized cells was calculated by the weight difference [31].

Scanning electron microscopy was conducted to verify yeast cell immobilization. Respectively, pieces of the immobilized biocatalyst were freeze dried as described previously. The samples were coated with gold in a Balzers SCD 004 Sputter coater (Bal-Tec, Schalksmühle, Germany) for 2 min and examined in a JSM-6300 scanning electron microscope (JEOL, Tokyo, Japan), operated at an accelerating voltage of 20 kV. Scanning electron micrographs were obtained at several magnifications. 

### 2.4. Successive Batch Fermentations of Various Sugars at Different Temperatures

The immobilized biocatalysts (100 g) were introduced into 400 mL of sterile synthetic liquid medium containing carbohydrates ~120 g L^−1^ (glucose, sucrose, or maltose) and successive batch fermentations were carried out without agitation at 28, 21, 14, and 7 °C. Towards the end of each batch, the fermentation liquid was decanted, and the immobilized cells were washed twice with a sterile synthetic medium (400 mL) for removal of free cells. Fermentations were monitored by determination of the °Be density at various time intervals and each system was submitted to fermentation until a final density of 0–0.5 °Be. For comparison reasons, successive batch fermentations with free yeast cells were also carried out at the same temperatures. Samples were collected at the end of fermentations and analyzed for ethanol, residual sugar, total phenolic content, and volatile by-products. 

### 2.5. Ethanol, Residual Sugar, and Major Volatiles Analyses 

Ethanol, residual sugar, and major volatiles (acetaldehyde, ethyl acetate, 1-propanol, isobutanol, methanol, and amyl alcohols) were determined as described by previous study [26]. Ethanol productivity was calculated as g of ethanol per liter liquid volume produced per day (g L^−1^ day^−1^). All analyses were conducted in triplicate (three independent samples) and the mean values are presented (max deviation for all values was about ±5%).

### 2.6. Phenolic Content Determination

The total phenol content of the fermented liquids was determined as described by previous study [32]. A calibration curve was obtained with gallic acid solutions (concentration range 50–500 mg L^−1^) and the results were expressed as gallic acid equivalents (GAE). All analyses were conducted in triplicate (three independent samples) and the mean values are presented (max deviation for all values was about ±5%).

### 2.7. Head space (HS) Solid Phase Microextraction (SPME) Gas Chromatography-Mass Spectrometry (GC-MS) Analysis

Samples of the produced alcoholic beverages were studied for volatile by-products composition and terpenoids content using HS-SPME GC-MS analysis. A total of 10 mL of each sample with 3.0 g NaCl were introduced into a 20 mL headspace vial fitted with a Teflon-lined septum sealed with an aluminum crimp seal, through which the SPME syringe needle (bearing a 2-cm fibre coated with 50/30 mm Divinylbenzene/Carboxen on poly-dimethyl-siloxane bonded to a flexible-fused silica core, Supelco, Bellefonte, PA, USA) was introduced. The container was then thermostated at 60 °C for 45 min. The absorbed volatile analytes were then analyzed by GC-MS (Shimadzu GC-17A, MS QP5050, capillary column Supelco CO Wax-10 60 m, 0.32 mm i.d., 0.25 μm film thickness). Helium was used as carrier gas at a flow rate of 1.8 mL/min. Oven temperature was set at 35 °C for 6 min, followed by a temperature gradient of 2 °C/min to 60 °C, held constant for 5 min, raised to 200 °C at 5 °C/min, and then to 250 °C at 25 °C/min with a final isothermal period of 6 min. The injector was operated in splitless mode. Injector and detector temperatures were 280 °C and 250 °C, respectively. The mass spectrometer was operated in the electron impact mode with the electron energy set at 70 eV and mass range *m*/*z* 29–400. Identification was performed by comparing the retention times with those of authentic compounds, by mass spectra of the authentic compounds generated in the laboratory (in-house libraries), by mass spectra obtained from NIST107, NIST21, and SZTERP libraries, and by determining kovats’ retention indexes and comparing with those reported in the literature. Kovats’ retention indexes (KI) were determined by injection of a standard mixture containing the homologous series of normal alkanes (C7–C32) in pure hexane under exactly the same experimental conditions, as described above. 4-Methyl-2-pentanol (Merck) diluted in pure ethanol was used as an internal standard (IS) at 1.62 mg L^−1^. The volatile compounds were semi-quantified by dividing the peak areas of the compounds of interest by the peak area of the IS and multiplying this ratio by the initial concentration of the IS (expressed as mg L^−1^). The peak areas were measured from the full scan chromatograph using total ion current (TIC). All analyses were carried out in triplicate (three independent samples) and the mean values are presented (standard deviation was about ±10% in most cases).

### 2.8. Preliminary Sensory Evaluation 

Samples of the produced alcoholic beverages were kept at 4 °C prior to sensory evaluation and tested regarding their aromatic characteristics. Sensory evaluation was conducted by 20 untrained tasters (wine consumers) familiar with wine tastes using the triangle test. Subsequently, the tasters were asked to give scores on a 0–10 scale using locally approved protocols in our laboratories regarding aroma characteristics [33]. Sensory evaluation was performed after 10, 30, 60, and 90 days of storage (4 °C) and was a blind test as the samples were coded by a different 3-digital number each and were served in a randomized order using a colored glass under no light. The evaluators were unaware of the sample’s identity. 

### 2.9. Experimental Design and Statistical Analysis

All fermentations were carried out in triplicate (three independent samples) and the mean values are presented (standard deviation for all values was about ±5% in most cases). The experiments were designed and analyzed statistically by ANOVA. Duncan’s multiple range test was used to determine significant differences among results. Coefficients and the ANOVA tables were computed using Statistica v.10 (StatSoft Inc., Tulsa, OK, USA). Principal component analysis (PCA) of data was computed using SPSS (v. 15.0, IBM Corp., Armonk, NY, USA).

## 3. Results and Discussion 

### 3.1. Yeast Cells Immobilization 

**Figure 1:**
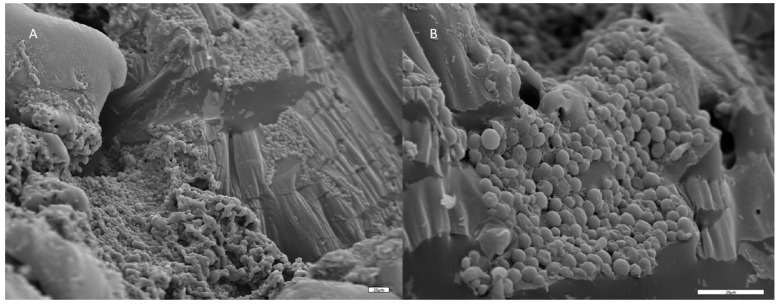
Electron micrographs of *Saccharomyces cerevisiae* AXAZ-1 immobilized on Pistacia terebinthus resin. A. magnification ×300, B: magnification ×1000.

Pieces of *P. terebinthus* resin were used as a cell immobilization carrier of *S. cerevisiae* AXAZ-1. The resin was used crude or melted (after heat treatment) and the yeast cells adhered well onto the surface of the carrier as illustrated in Figure 1. 

The immobilized biocatalysts were used in successive fermentation batches of glucose synthetic medium at 28 and 21 °C in order to identify the optimum conditions for alcoholic fermentation. The immobilized biocatalyst prepared by the use of crude resin pieces resulted in lower (*p* < 0.05) fermentation times at both temperatures and thus, was selected for further experiments. 

### 3.2. Successive Fermentations Batches by Various Sugars at Different Temperatures

The immobilized biocatalysts were used in successive batch fermentations using a synthetic medium that contained ~120 g L^−1^ of glucose, sucrose, and maltose, respectively (Table 1). Batch fermentations were also tested at different temperatures (28, 21, 14, and 7 °C). In all cases, batch fermentations were conducted by the use of free cells for comparison reasons under the same conditions. 

Fermentation kinetics proved to be more efficient in the case of immobilized cells providing lower fermentation times (*p* < 0.05) compared with free cells (Table 1). Regarding the fermentation temperature, the immobilized biocatalysts proved to achieve better kinetics in temperatures of 28, 21, and 14 °C, while increased fermentation values were observed at 7 °C. In all cases, fermentation kinetics remained in levels accepted by the industry. Similar increase in fermentation times at low temperatures has also been reported before by the psychrophilic strain AXAZ-1 [34,35] or by adaptation of the *Saccharomyces cerevisiae* species [36]. In the present study, the fermentation times at low temperature (7 °C) proved to be slightly increased in the case of the immobilized yeast cells on *P. terebinthus* resin when compared to immobilized cells of the same strain on other natural supports [26,37,38]. A possible explanation to this outcome could be the hardening of the resin at a low temperature of 7 °C, which might result in diffusion difficulties between fermentation substrates and products. According to other studies, Pistacia resins are reported to provide antimicrobial properties [2,4,39] and as a result, yeasts lower populations and increased fermentation times of the present study could be a consequence of the resins antimicrobial characteristics. Nevertheless, fermentations accomplished by the use of the novel immobilized biocatalyst proved to be successful despite the possible antimicrobial effects of the resin. 

Ethanol production remained at similar levels in fermented beverages produced by immobilized or free cells. However, ethanol productivity in fermentations carried out by immobilized cells was higher (*p* < 0.05) than free cells at all temperatures (Table 1), as fermentation time was significantly lower in the case of the immobilized biocatalyst. 

### 3.3. Formation of Major Volatile By-Products

Since the fermentation products are designated for potable alcohol production, which is usually added in various kinds of alcoholic drinks, such as liqueurs, sweet wines, and distillates, a study on the formation of major volatiles was conducted (Table 2). Acetaldehyde, ethyl acetate, 1-propanol, isobutanol, and amyl alcohols are the major volatile by-products produced during fermentation and accounted for more than half of the total volatiles [40]. In order to assess the contribution of the volatiles produced during fermentation and those derived from the immobilization support (*P. terebinthus* resin) on the aroma and taste of the produced alcoholic liquids, an examination of fermented beverages for volatile by-products was carried out by both immobilized and free cells (Table 2). 

Aldehydes are the oxidation products of alcohols and are usually detected at very low levels in alcoholic beverages [41]. Acetaldehyde specifically, can provide a pleasant fruity aroma when it is detected at low concentration. On the other hand, acetaldehyde can give a pungent, undesirable, irritating odor to the product when it is present at higher levels. Hence, it is crucial, especially in the case of potable alcohol, that sugar fermentation is performed by yeast strains that produce low amounts of acetaldehyde [40]. In the present study, the detected acetaldehyde concentration ranged within acceptable levels [42]. In most cases, acetaldehyde ranged in higher concentrations in glucose and sucrose fermented mediums compared to maltose while no effect was reported by the use of the starter culture (free or immobilized) (*p* > 0.05) in the case where glucose and maltose-based media were used.

Ethyl acetate, the most important and abundant ester in wines, ranged in concentrations from 3.2 to 10.3 mg L^−1^. *Saccharomyces cerevisiae* strains usually produce intermediate levels of ethyl acetate while its concentration is desirable in levels between 120–150 mg L^−1^, providing fruity notes, while at higher concentrations is considered unpleasant and may result in a spoiled, pungent tang aroma bouquet [43,44].

Higher alcohols are considered the largest group of flavor compounds in wines and fermented liquids [41]. Among them, 1-propanol, isobutanol, and amyl alcohols are considered crucial for aroma quality [43]. In the current study, concentrations of 1-propanol, isobutanol, and amyl alcohols were up to 3 mg L^−1^, 4.2 mg L^−1^, and 19.7 mg L^−1^, respectively. The low concentration of higher alcohols is an indication of high quality in wines and in fermented liquids, since high concentrations are related to off flavors. The methanol content ranged 12.9–50.4 mg L^−1^, indicating the quality of the fermented products. Due to its toxic properties, concentrations >0.1–0.2 g L^−1^ are undesirable [43].

In addition, it has already been established that phenolic compounds contribute to the sensory characteristics of alcoholic beverages [45]. In the present study, the polyphenol content was significantly affected (*p* < 0.05) by the fermentation temperature. Likewise, fermentations carried out by the immobilized biocatalyst resulted in significantly (*p* < 0.05) higher content of polyphenols (Table 2), while increased amounts were recorded between 21–28 °C. These results agree with previous studies reporting that the extracted quantity of polyphenols can be affected by the fermentation temperature and the solvent type [46]. In addition, the presence of polyphenols can provide an antioxidant effect to the product. Hence, their present is desirable in alcoholic beverages which are produced without the addition of preservatives [47,48]. The stability and content of polyphenols can also be affected by pH, metal ions, exposure to light, storage time, oxygen, temperature, and enzymatic activities [49].

Propanol and isobutanol were also detected in limited concentrations and the results showed that their presence within the fermented liquid was significantly affected by the fermentation temperature (*p* < 0.05). 

Overall, based on the above results, all products were considered of acceptable quality. 

### 3.4. SPME GC-MS Analysis

Even though the chemical composition of the *P. terebinthus* resin essential oils has been studied [50], the effect of *P. terebinthus* resin on the volatile composition of fermented liquids has never been reported before, as only triterpenes of the galls of the resin have been elucidated [51]. In the present study, HS-SPME GC-MS technique was used to determine the volatile compounds in fermentations carried out by immobilized cells of *S. cerevisiae* AXAZ-1 on *P. terebinthus* resin, in comparison to fermentations by free cells, at 7, 14, 21, and 28 °C.

Qualitative results of the volatile compounds are presented in Table 3. In total, 147 compounds were detected and identified in the alcoholic liquids fermented by immobilized or free cells. The compounds included mainly esters, organic acids, alcohols, carbonyl compounds, and terpenes. 

Since the main aim of the study was to assess the potential extraction of terpenoides from *Pistacia terebintus* resin into the new products, analysis of the volatile compounds was focused on the identified terpenes. Monoterpenes, oxygenated monoterpenes, and sesquiterpenes were mainly detected. Several terpenes, such as *α*-pinene, verbenyl ethyl ether, linalool, dehydro-*p*-cymene, bornyl acetate, 4-terpineol, L-*trans*-pinocarveol, *α*-phellandren-8-ol, borneol, verbenol, exo-hydroxycineol, verbenone, *p*-methyl-acetophenone, myrtenol, and *trans*-carveol were only detected in fermentations carried by the immobilized cells at all temperatures. However, the number of terpenes found in alcoholic liquids fermented by the immobilized cells decreased with the fermentation temperature (Table 3). Thus, several terpenes, such as carvomenthol, *trans*-pinane, 1,8-cineole, *p*-menth-1-en-9-al, *p*-cymenene, *δ*-terpineol, neral, geraniol, geranyl acetate, *p*-cymen-8-ol, *p*-cymen-9-ol, *trans*-myrtanol, piperitenone, *β*-camphor, *p*-cresol, cuminyl alcohol, pseudoionone, longipinanol, and farnesol were only identified in the alcoholic liquids produced by the immobilized cells at temperatures ≥21 °C. On the other hand, camphene and thuja-2,4(10)-diene were only present in fermented products at 7 °C, but not at 28, 21, and 14 °C, probably due to vaporization at room temperature. 

From a quantitative point of view, significantly (*p* < 0.05) higher amounts of terpenes were detected in fermentations with immobilized cells in all cases, as expected (Table 4). The content of terpenes also seemed to depend on the fermented sugar. Thus, the highest (*p* < 0.05) concentration of terpenes was observed in sucrose fermentation at 7 °C (579.01 mg L^−1^). On the contrary, a decrease was noticed at temperatures <28 °C in glucose and maltose fermentations.

### 3.5. Chemometrics

Principal component analysis is used in exploratory analysis, as it gives graphical representations of inter-sample and inter-variable relationships and provides a way to reduce the complexity of the data.

**Figure 2:**
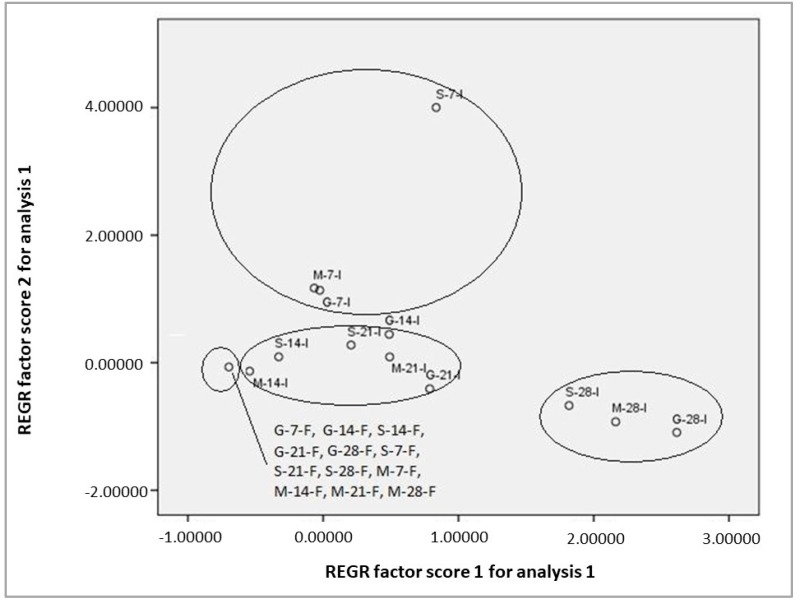
Principal component analysis (PCA) plot of terpenoids in fermentation batches of synthetic mediums by free or immobilized *S. cerevisiae* AXAZ-1 cells on *Pistacia terebinthus* resin at different temperatures. (The numbers in the sample codes indicate the fermentation temperature. G: glucose synthetic medium, S: Sucrose synthetic medium, M: maltose synthetic medium. I: Immobilized *Saccharomyces cerevisiae* AXAZ-1 cells on *Pistacia terebinthus* resin, F: Free cells of *Saccharomyces cerevisiae* AXAZ-1).

The application of the PCA algorithm to data concerning terpenes content showed four distinctive groups (Figure 2). The first group was composed by liquids fermented by immobilized cells at 28 °C, while the second group by samples fermented by immobilized cells at 21 and 14 °C. A third group consisting of liquids fermented by immobilized cells at 7 °C was evident. Finally, the fourth group contained all samples fermented with free cells projected at almost the same point, as the content of terpenes ranged in similar (low) levels. 

Hence, the results indicated that primarily the nature of the cells (immobilized or free) and the fermentation temperature affected the terpenes’ composition.

### 3.6. Phenolic Content 

Fermentations carried out by immobilized cells resulted in a significantly (*p* < 0.05) higher content of polyphenols. More specifically, their average content in the case of free cells was 24.3–70.2 mg GAE L^−1^ in glucose synthetic medium fermentations, 34.3–49.3 mg GAE L^−1^ in sucrose fermentations, and 30.2–61.8 mg GAE L^−1^ in maltose fermentations, while in the case of immobilized cells the average content was 74.3–171.8 mg GAE L^−1^, 66.0-109.3 mg GAE L^−1^, and 63.6–113.5 mg GAE L^−1^, respectively (Table 2). 

**Figure 3:**
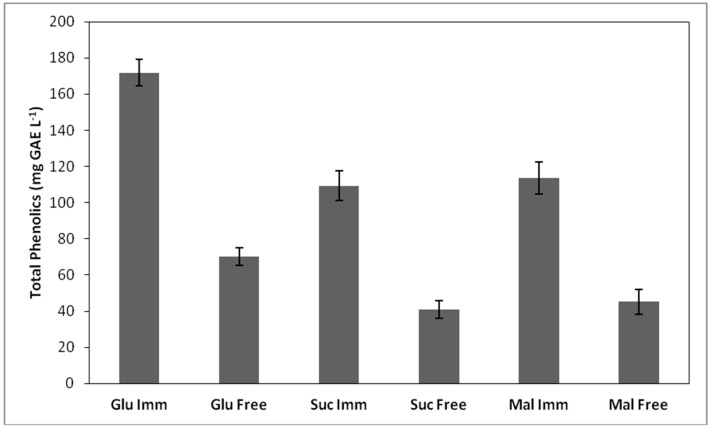
Total phenolic content (mg GAE L^−1^) of the produced alcoholic beverages at 21 °C.

The extracted polyphenols content was significantly affected (*p* < 0.05) by the fermentation temperature, while the highest polyphenol content was observed in fermentations carried out at 21 °C, in the case of immobilized cells (Figure 3). The results agree with a previous study reporting that the extracted quantity of polyphenols can be affected by the fermentation temperature and the solvent type [46]. 

### 3.7. Preliminary Sensory Evaluation and Resistance to Spoilage

The use of immobilized cells on *P. terebinthus* resin provided a distinctive aromatic character to the fermented beverages, which was also detected throughout the whole storage period (90 days). The capacity of *P. terebinthus* resin to provide unique aromatic characteristics to fermented products has also been reported by previous studies [2,13]. Regarding sensory evaluation of the current study, the panel showed a high preference (*p* < 0.05) on the fermented beverages produced by the immobilized cells (scores 7.42 ± 0.96) compared to the beverages produced by free cells (scores 5.21 ± 1.09).

It is noteworthy that the fermented alcoholic beverages remained for 90 days at room temperature without any treatment (e.g., addition of preservatives) and no contamination or spoilage was macroscopically observed. On the other hand, the fermented alcoholic beverages produced by free cells showed spoilage affects mainly attributed to overgrowth of white and green molds on the surface of the fermented liquids. Similar results were also reported by Schoina, Terpou, Angelika-Ioanna, Koutinas, Kanellaki, and Bosnea [13] showing an improved resistance against spoilage microorganisms in yoghurts produced with incorporated *P. terebintus* resin. This result may be attributed to the high antimicrobial and antioxidant effect of the resin deriving mainly from the high terpene content. Specifically, many of the terpenes detected in the fermented beverages produced by the immobilized biocatalyst have shown considerable antimicrobial and antioxidant activities. Kotan et al. reported a variable degree of antibacterial activities of *α*-terpineol, borneol, carvone, linalool, 4-terpineol, bornyl acetate, 1,8-cineole, and geranyl acetate [52], while Duru et al. observed antifungal activity of total and neutral fractions of essential oil obtained from the *Pistacia lentiscus* resin against *R. solani* [7]. Similarly, farnesol displayed antimicrobial action against *Staphylococcus aureus* [53] and *Staphylococcus epidermidis* [54].

## 4. Conclusions

*P. terebinthus* resin proved to be a suitable immobilization support of yeast cells resulting in successive alcoholic fermentations. In addition, this novel immobilized biocatalyst can be used for alcoholic fermentations in a wide range of temperatures. The terpene and total phenolic content detected in alcoholic beverages and deriving from the immobilized biocatalyst may potentially contribute to antioxidant, antibacterial, and antifungal properties of the fermented alcoholic beverages. Finally, the use of *P. terebinthus* resin as yeasts immobilization support has high commercialization potential in the beverages sector providing unique aromatic and low alcohol content products with a low or negligible amount of preservatives. 

## Figures and Tables

**Table 1:** Kinetic parameters during fermentation batches of the synthetic medium using free or immobilized yeast cells on *P. terebinthus* resin at various temperatures.

	Batch No.	T (°C)	Fermentation Time (h)	Ethanol (g L^−1^)	Ethanol Productivity (g L^−1^ Day^−1^)	Residual Sugar (g L^−1^)	Sugar Conversion (%)
Free cells	Glucose						
	1−8	28	29.0 ± 3.2	43.45 ± 5.5	35.9 ± 1.7	3.4 ± 1.2	97.5 ± 2.5
	9−13	21	42.0 ± 3.7	40.29 ± 5.5	19.9 ± 2.3	3.3 ± 1.5	98.2 ± 1.7
	14−16	14	133.3 ± 6.1	36.34 ± 3.9	9.0 ± 0.3	5.9 ± 0.7	95.3 ± 0.3
	17−18	7	1140 ± 84.9	32.39 ± 2.4	0.7 ± 0.1	22.3 ± 2.1	83.1 ± 0.2
	Sucrose						
	1−8	28	32.1 ± 2.9	46.60 ± 5.5	34.8 ± 5.6	19.9 ± 6.4	85.0 ± 4.9
	9−13	21	60.2 ± 2.6	40.30 ± 5.5	16.2 ± 2.4	20.5 ± 1.9	84.5 ± 1.5
	14−16	14	146 ± 16.7	36.34 ± 3.9	6.0 ± 1.3	20.9 ± 4.6	84.2 ± 3.5
	17−18	7	1520 ± 84.9	41.08 ± 2.4	0.7 ± 0.1	22.4 ± 3.3	83.0 ± 2.5
	Maltose						
	1−8	28	26.1 ± 1.4	46.61 ± 5.5	42.5 ± 4.8	3.1 ± 1.0	97.6 ± 4.0
	9−13	21	50.8 ± 3.1	32.40 ± 3.2	15.3 ± 0.9	5.4 ± 3.5	96.1 ± 2.8
	14−16	14	167.3 ± 14.6	32.39 ± 2.4	4.7 ± 0.7	13.3 ± 3.1	90.1 ± 2.0
	17−18	7	2160 ± 99.0	41.10 ± 12.6	0.5 ± 0.1	16.1 ± 1.3	88.3 ± 0.4
Immobilized cells	Glucose						
	1−8	28	19.6 ± 2.7	37.30 ± 2.4	45.8 ± 4.7	9.4 ± 6.5	93.0 ± 5.4
	9−13	21	23.9 ± 3.5	37.30 ± 1.6	37.5 ± 5.7	10.1 ± 2.3	92.7 ± 7.1
	14−16	14	29.9 ± 1.9	45.03 ± 2.4	36.4 ± 1.9	9.1 ± 3.4	93.1 ± 4.2
	17−18	7	376 ± 24.0	39.50 ± 0.8	2.6 ± 0.2	20.3 ± 3.3	84.7 ± 2.3
	Sucrose						
	1−8	28	24.2 ± 4.3	42.66 ± 6.3	43.4 ± 8.0	20.6 ± 11.8	84.4 ± 8.9
	9−13	21	33.3 ± 4.6	42.66 ± 3.9	30.9 ± 4.6	17.2 ± 6.3	86.9 ± 4.8
	14−16	14	53.5 ± 1.0	44.24 ± 4.7	19.9 ± 1.8	13.9 ± 10.5	89.5 ± 9.0
	17−18	7	705 ± 59.8	50.56 ± 2.4	1.7 ± 0.2	13.6 ± 1.4	89.5 ± 0.9
	Maltose						
	1−8	28	24.0 ± 3.9	39.50 ± 4.7	39.7 ± 7.6	21.1 ± 6.2	84.1 ± 4.8
	9−13	21	22.7 ± 4.5	41.90 ± 3.9	45.6 ± 5.6	15.4 ± 5.8	89.1 ± 4.9
	14−16	14	34.7 ± 3.3	37.13 ± 5.5	25.8 ± 6.0	5.3 ± 2.4	96.4 ± 2.4
	17−18	7	792 ± 30.2	34.76 ± 1.6	1.0 ± 0.1	21.3 ± 4.3	83.8 ± 2.7

**Table 2:** The effect of fermentation temperatures on volatile by-products and polyphenols content during successive fermentation batches of synthetic mediums using free or immobilized yeast cells on *P. terebinthus* resin.

Substrate	Batch No.	T (°C)	MeOH (mg L^−1^)	Acetaldehyde (mg L^−1^)	Ethyl Acetate (mg L^−1^)	Propanol (mg L^−1^)	Isobutyl Alcohol (mg L^−1^)	Amyl Alcohols (mg L^−1^)	Total Polyphenols (mg GAE L^−1^)
Glucose	Free cells								
	1−8	28	16.3 ± 6.0 ^e,a,b,f,g^	49.8 ± 14.6 ^e,a,d^	9.9 ± 1.7 ^e,a,d,f,h^	Tr ^a^	2.9 ± 0.3 ^e^	15.8 ± 2.6 ^e,a,b,d,g,h^	48.5 ± 4.3 ^e,g^
	9−13	21	12.9 ± 3.6 ^f,a,b,e^	32.0 ± 11.2 ^f,a,b,c,d,g,h^	8.6 ± 1.9 ^f,a,b,c,d,e,h^	0.5 ± 0.2 ^c^	1.0 ± 0.3 ^f,b,c,d^	5.3 ± 1.5 ^f,c^	70.2 ± 4.7 ^f,d^
	14−16	14	24.1 ± 7.3 ^g,a,b,c,d,e^	18.4 ± 3.7 ^g,b,c,f,h^	5.6 ± 1.1 ^g,a,b,c^	1.3 ± 0.6 ^b^	4.3 ± 0.7 ^g,a,h^	13.8 ± 2.6 ^g,b,c,d,e,h^	47.1 ± 4.7 ^g,e^
	17−18	7	46.9 ± 10.6 ^h^	23.0 ± 3.2 ^h,a,b,c,f,g^	10.3 ± 3.5 ^h,a,d,e,f^	Tr ^a^	4.8 ± 1.2 ^h,a,g^	18.2 ± 3.9 ^h,a,d,e,g^	24.3 ± 4.7 ^h^
	Immobilized cells								
	1−8	28	21.1 ± 5.8 ^a,b,c,e,f,g^	37.0 ± 10.1 ^a,b,c,d,e,f,h^	7.5 ± 1.1 ^a,b,c,d,e,f,g,h^	Tr ^a^	4.2 ± 0.8 ^a,g,h^	19.7 ± 4.1 ^a,d,e,h^	169.3 ± 6.8 ^a,b^
	9−13	21	21.7 ± 5.3 ^b,a,c,e,f,g^	20.8 ± 2.2 ^b,a,c,f,g,h^	6.9 ± 1.2 ^b,a,c,f,g^	1.0 ± 0.4 ^b^	1.7 ± 0.9 ^b,d,f^	10.7 ± 2.2 ^b,c,d,e,g^	171.8 ± 7.3 ^b,a^
	14−16	14	28.5 ± 6.7 ^c,a,b,d,g^	23.5 ± 4.4 ^c,a,b,d,f,g,h^	6.3 ± 1.4 ^c,a,b,f,g^	Tr ^a^	Tr ^c,d,f^	9.8 ± 3.1 ^c,b,f,g^	104.3 ± 5.5 ^c^
	17−18	7	31.9 ± 7.1 ^d,c,g^	40.6 ± 7.3 ^d,a,c,e,f^	9.9 ± 2.9 ^d,a,e,f,h^	Tr ^a^	0.9 ± 0.3 ^d,b,c,f^	15.5 ± 3.7 ^d,a,b,e,g,h^	74.3 ± 4.3 ^d,f^
Sucrose	Free cells								
	1−8	28	33.5 ± 7.5 ^e,a,f,g^	38.9 ± 7.1 ^e,d,f,h^	3.2 ± 1.1 ^e,a,c,d,g,h^	Tr ^d^	5.0 ± 1.4 ^e,a^	15.5 ± 4.9 ^a^	40.2 ± 5.5 ^e,f,g,h^
	9−13	21	36.0 ± 8.8 ^f,a,e,g^	30.7 ± 7.3 ^f,a,b,d,e,g^	9.7 ± 4.5 ^f,b^	Tr ^d^	Tr ^f,c,d,g,h^	Tr ^b^	41.0 ± 4.9 ^f,e,g,h^
	14−16	14	39.7 ± 7.4 ^g,d,e,f,h^	25.1 ± 6.7 ^g,a,b,c,f^	Tr ^g,e^	Tr ^d^	Tr ^g,c,d,f,h^	Tr ^b^	49.3 ± 5.4 ^g,e,f^
	17−18	7	50.4 ± 11.4 ^h,d,g^	44.3 ± 11.3 ^h,d,e^	4.5 ± 1.2 ^h,a,c,d,e^	Tr ^d^	1.2 ± 0.6 ^h,b,c,d,f,g^	Tr ^b^	34.3 ± 4.7 ^h,e,f^
	Immobilized cells								
	1−8	28	24.7 ± 5.1 ^a,f^	24.0 ± 5.3 ^a,b,c,f,g^	5.0 ± 1.7 ^a,c,d,e,h^	1.6 ± 0.6 ^a,b,c^	4.2 ± 1.4 ^a,b,e^	13.3 ± 2.2 ^a^	101.8 ± 7.1 ^a,b^
	9−13	21	21.3 ± 3.7 ^b,a,c^	21.3 ± 2.9 ^b,a,c,f,g^	8.9 ± 1.4 ^b,c,f^	1.3 ± 0.5 ^b,a^	2.7 ± 1.1 ^b,a,c,h^	15.1 ± 3.3 ^a^	109.3 ± 8.2 ^b,a^
	14−16	14	19.2 ± 3.5 ^c,a^	15.2 ± 6.0 ^c,a,b,g^	5.9 ± 1.4 ^c,a,b,d,e,h^	2.3 ± 1.1 ^c,a^	1.2 ± 0.3 ^c,b,d,f,g,h^	11.3 ± 2.9 ^a^	88.5 ± 5.9 ^c^
	17−18	7	48.3 ± 11.1 ^d,g,h^	37.9 ± 5.8 ^d,e,f,h^	4.0 ± 1.2 ^d,a,c,e,h^	Tr ^d^	0.4 ± 0.1 ^d,c,f,g,h^	Tr ^b^	66.0 ± 5.1 ^d^
Maltose	Free cells								
	1−8	28	15.5 ± 3.4 ^e,a,c,f^	15.3 ± 4.7 ^e,a^	4.2 ± 2.1 ^a^	2.4 ± 0.8 ^e,a,b,c,f^	3.5 ± 1.0 ^e,a,c,f^	14.7 ± 3.2 ^e,a,b,c,d^	61.8 ± 5.1 ^e,c,d^
	9−13	21	14.5 ± 4.9 ^f,a,c,e^	23.3 ± 6.0 ^f,b^	6.0 ± 3.8 ^a^	1.2 ± 0.4 ^f,b,c,d,e,g,h^	1.7 ± 1.7 ^f,b,c,d,e,g,h^	5.6 ± 5.2 ^f,a,b,c,g,h^	45.2 ± 6.8 ^f^
	14−16	14	31.1 ± 4.2 ^g,b,d,f,h^	4.5 ± 1.2 ^g,c,d,h^	5.6 ± 1.3 ^a^	Tr ^g,b,c,d,f,h^	Tr ^g,d,f,h^	Tr ^g,f,h^	30.2 ± 3.9 ^g,h^
	17−18	7	26.5 ± 4.6 ^h,b,c,g^	5.1 ± 1.3 ^h,c,d,g^	5.0 ± 1.4 ^a^	Tr ^h,c,d,f,g^	Tr ^h,d,f,g^	Tr ^h,f,g^	32.7 ± 5.3 ^h,g^
	Immobilized cells								
	1−8	28	17.1 ± 5.8 ^a,c,e,f^	16.0 ± 2.9 ^a,e^	5.8 ± 1.8 ^a^	3.0 ± 1.5 ^a,b,e^	4.0 ± 1.0 ^a,b,c,e^	13.3 ± 4.6 ^a,b,c,d,e^	96.8 ± 7.7 ^a^
	9−13	21	26.2 ± 5.7 ^b,c,g,h^	27.2 ± 4.9 ^b,f^	8.0 ± 1.9 ^a^	1.6 ± 1.0 ^b,a,c,d,e,f,g^	2.5 ± 1.3 ^b,a,c,e,f^	9.4 ± 4.1 ^b,a,c,e,f^	113.5 ± 8.9 ^b^
	14−16	14	21.2 ± 4.2 ^c,a,b,e,h^	2.4 ± 0.5 ^c,d,g,h^	7.3 ± 1.1 ^a^	0.8 ± 0.2 ^c,b,d,e,f,g,h^	2.5 ± 1.0 ^c,a,b,e,f^	10.7 ± 2.3 ^c,a,b,e,f^	71.8 ± 5.1 ^c,d,e^
	17−18	7	36.4 ± 6.1 ^d^	6.0 ± 1.1 ^d,c,g,h^	3.9 ± 1.0 ^a^	Tr ^d,b,c,f,g,h^	Tr ^d,f,g,h^	18.7 ± 5.6 ^d,a,e^	63.6 ± 5.4 ^d,c,e^

Two-way ANOVA was performed to study the effect of cell immobilization and fermentation temperature on volatile by-products and total phenolics. Significant differences for fermentations carried out with the same sugar source are shown with different letters in superscript.

Tr: Traces; GAE: gallic acid equivalents.

**Table 3:** Volatile compounds detected by solid phase microextraction/gas chromatography-mass spectrometry (SPME/GC-MS) analysis in fermentation batches of synthetic mediums by free or immobilized *S. cerevisiae* AXAZ-1 cells on *P. terebinthus* resin at different temperatures.

Compound	28 °C						21 °C						14 °C						7 °C						Total Compounds Detected
	GI	GF	SI	SF	MI	MF	GI	GF	SI	SF	MI	MF	GI	GF	SI	SF	MI	MF	GI	GF	SI	SF	MI	MF	
Alcohols	50	18	44	13	41	18	41	17	29	17	32	16	37	14	26	13	33	15	24	15	24	13	26	14	57
terpenoids	27	2	26	1	25	2	21	1	16	2	17	2	18	2	13	1	14	2	11	1	11	1	11	1	30
Esters	16	9	15	10	12	13	15	11	16	11	16	13	13	6	16	9	11	9	16	10	15	10	16	10	24
terpenoids	3	1	2	-	2	1	3	1	3	1	3	1	2	1	2	-	2	1	2	1	2	1	2	1	3
Organic acids	10	9	10	10	9	10	10	10	9	7	7	9	2	4	5	5	2	3	4	2	6	4	2	4	10
terpenoids	-	-	-	-	-	-	-	-	-	-	-	-	-	-	-	-	-	-	-	-	-	-	-	-	-
Aldehydes	6	2	3	2	5	1	11	3	9	2	12	1	4	1	5	3	3	1	2	1	3	1	3	1	13
terpenoids	4	-	2	-	4	-	3	-	2	-	3	-	2	-	2	-	2	-	2	-	2	-	2	-	4
Ketones	13	6	17	4	15	6	13	7	10	7	12	6	13	8	10	5	10	8	12	7	10	7	10	6	21
terpenoids	8	-	8	-	8	-	6	-	3	-	5	-	5	-	3	-	3	-	4	-	4	-	4	-	9
Miscellaneous compounds	16	10	18	7	15	9	16	10	18	8	19	8	12	5	12	8	13	10	16	10	14	10	15	11	22
terpenoids	8	1	8	-	8	1	7	1	8	-	8	1	4	-	4	-	5	1	7	1	7	1	7	1	11
Total terpenoids	50	4	46	1	47	4	40	3	32	3	36	4	31	3	24	1	26	4	26	3	26	3	26	3	57
Total compounds	111	54	107	46	97	57	106	58	91	52	98	53	81	38	74	43	72	46	74	45	72	36	72	46	147

G: glucose synthetic medium, S: Sucrose synthetic medium, M: maltose synthetic medium. I: Immobilized *Saccharomyces cerevisiae* AXAZ-1 cells on *Pistachia terebinthus* resin, F: Free cells of *Saccharomyces cerevisiae* AXAZ-1.

**Table 4:** Semi-quantification of terpenoids (mg L^−1^) detected by SPME/GC-MS analysis in fermentation batches of synthetic mediums by free or immobilized *S. cerevisiae* AXAZ-1 cells on *P. terebinthus* resin at different temperatures.

Compounds	KI	G7-I	G7-F	G14-I	G14-F	G21-I	G21-F	G28-I	G28-F	S7-I	S7-F	S14-I	S14-F	S21-I	S21-F	S28-I	S28-F	M7-I	M7-F	M14-I	M14-F	M21-I	M21-F	M28-I	M28-F
p-menth-4(8)-ene	946	23.90	18.36	-	-	24.86	18.57	1.18	5.45	25.00	1.17	1.46	-	1.24	-	1.66	-	1.84	0.97	0.81	1.14	1.28	-		
α-pinene	994	23.30	-	47.47	-	51.39	-	7.79	-	386.54	-	1.92	-	14.35	-	9.98	-	1.90	-	4.83	-	13.07	-	36.24	-
carvomenthol	1024	-	-	-	-	-	-	5.38	-	-	-	-	-	-	-	4.84	-	-	-	-	-	-	-	9.30	-
camphene	1033	1.93	-	-	-	-	-	-	-	6.50	-	-	-	-	-	-	-	5.86	-	-	-	-	-	-	-
trans-pinane	1038	-	-	-	-	-	-	15.17	-	-	-	-	-	15.06	-	1.16	-	-	-	-	-	0.40	-	0.65	-
β-pinene	1085	0.52	-	-	-	0.13	-	0.06	-	8.79	-	-	-	0.93	-	1.03	-	3.77	-	0.12	-	0.54	-	0.33	-
thuja-2,4(10)-diene	1119	0.41	-	-	-	-	-	-	-	2.60	-	-	-	-	-	-	-	1.19	-	-	-	-	-	-	-
1,8-cineole	1205	-	-	-	-	0.27	-	5.19	-	-	-	-	-	0.33	-	1.09	-	-	-	-	-	0.53	-	0.75	-
p-menth-1-en-9-al	1235	-	-	-	-	0.24	-	3.70	-	-	-	-	-	-	-	0.33	-	-	-	-	-	4.48	-	3.71	-
verbenyl ethyl ether	1333	4.06	-	4.80	-	0.20	-	5.30	-	15.09	-	0.31	-	1.11	-	0.55	-	0.76	-	0.29	-	2.02	-	1.43	-
p-cymenene	1410	-	-	-	-	0.24	-	0.27	-	-	-	-	-	0.35	-	1.09	-	-	-	-	-	1.05	-	2.88	-
dehydro-p-cymene	1438	4.96	-	2.68	-	3.55	-	3.42	-	14.94	-	1.53	-	199.91	-	5.95	-	9.38	-	0.62	-	2.37	-	18.81	-
linalool oxide	1464	-	-	2.13	-	0.85	-	0.51	-	-	-	-	-	0.39	-	0.43	-	-	-	0.21	-	0.33	-	0.29	-
α-campholenal	1484	1.21	-	0.09	-	0.17	-	0.15	-	1.46	-	0.31	-	0.31	-	-	-	0.18	-	0.24	-	1.41	-	0.78	-
pinocarvone	1521	2.01	-	2.58	-	-	-	2.39	-	5.46	-	-	-	1.27	-	1.73	-	39.62	-	0.25	-	1.05	-	2.95	-
α-norborneol	1525	-	-	-	-	-	-	0.34	-	-	-	-	-	-	-	0.23	-	-	-	-	-	-	-	-	-
linalool	1536	1.21	-	2.69	-	0.15	-	0.97	-	1.35	-	0.95	-	1.09	-	0.32	-	0.58	-	0.13	-	0.66	-	0.60	-
fenchol	1541	-	-	4.79	-	1.88	-	2.39	-	-	-	1.06	-	0.90	-	1.13	-	-	-	0.35	-	1.93	-	2.95	-
bornyl acetate	1550	5.98	-	6.10	-	0.63	-	0.86	-	15.65	-	1.04	-	2.00	-	0.94	-	0.90	-	0.33	-	1.57	-	1.44	-
isopinocamphone	1557	-	-	1.56	-	-	-	1.56	-	-	-	11.81	-	-	-	0.83	-	-	-	-	-	0.69	-	0.97	-
4-terpineol	1562	2.10	-	3.47	-	2.48	-	5.83	-	3.18	-	1.21	-	1.25	-	4.88	-	5.17	-	0.36	-	2.76	-	3.71	-
myrtenal	1588	2.41	-	0.72	-	0.55	-	0.35	-	7.03	-	0.49	-	1.59	-	-	-	0.19	-	0.42	-	1.49	-	2.69	-
3,6,6-trimethyl-2-norpinanone	1595	-	-	1.13	-	0.62	-	-	-	-	-	-	-	-	-	-	-	-	-	-	-	-	-	1.09	-
L-trans-pinocarveol	1618	3.41	-	2.23	-	2.00	-	5.70	-	7.74	-	0.12	-	2.50	-	4.64	-	3.18	-	0.31	-	2.79	-	4.85	-
α-phellandren-8-ol	1626	0.86	-	0.93	-	0.41	-	1.27	-	3.45	-	4.62	-	3.97	-	5.83	-	1.46	-	0.17	-	1.29	-	1.73	-
δ-terpineol	1636	-	-	-	-	-	-	0.11	-	-	-	-	-	-	-	0.16	-	-	-	-	-	-	-	0.14	-
bornylene	1647	-	-	0.51	-	-	-	-	-	-	-	-	-	-	-	-	-	-	-	-	-	-	-	-	-
neral	1652	-	-	-	-	-	-	0.50	-	-	-	-	-	-	-	0.24	-	-	-	-	-	-	-	0.34	-
α-terpineol	1665	8.32	-	11.89	0.48	17.40	-	34.65	0.43	15.09	-	8.50	-	6.92	0.52	23.67	-	7.08	-	3.96	0.23	17.96	1.16	22.93	0.66
borneol	1668	0.80	-	1.28	-	2.72	-	2.61	-	1.22	-	1.99	-	1.86	-	2.10	-	2.82	-	0.87	-	5.76	-	29.15	-
isopinocampheol	1675	-	-	1.20	-	-	-	-	-	-	-	-	-	-	-	-	-	-	-	-	-	-	-	-	-
verbenol	1679	3.59	-	1.84	-	0.84	-	1.59	-	16.76	-	0.87	-	1.29	-	2.36	-	1.17	-	0.78	-	0.61	-	0.52	-
endo,endo-2,3-bornanediol	1688	-	-	0.41	-	0.20	-	0.23	-	-	-	-	-	0.17	-	0.21	-	-	-	-	-	-	-	0.23	-
exo-hydroxycineol	1696	0.97	-	2.14	-	2.82	-	6.65	-	1.85	-	0.63	-	6.79	-	5.00	-	0.22	-	0.28	-	1.91	-	2.29	-
myrcenol	1696	-	-	-	-	-	-	-	-	-	-	-	-	1.41	-	-	-	-	-	-	-	-	-	-	-
verbenone	1705	4.58	-	2.53	-	2.73	-	4.64	-	17.00	-	0.87	-	2.96	-	3.94	-	4.82	-	1.18	-	0.31	-	0.14	-
carvone	1707	0.56	-	-	-	0.21	-	0.56	-	1.33	-	-	-	-	-	0.62	-	0.37	-	-	-	0.54	-	0.85	-
p-methyl-acetophenone	1754	1.55	-	1.79	-	1.71	-	2.07	-	3.84	-	1.19	-	2.02	-	2.19	-	2.81	-	0.71	-	2.94	-	2.88	-
β-phellandren-8-ol	1757	-	-	0.47	-	0.71	-	1.50	-	-	-	-	-	-	-	0.77	-	-	-	-	-	0.31	-	0.59	-
myrtenol	1767	5.59	-	3.20	-	3.95	-	3.54	-	8.70	-	1.05	-	2.90	-	3.69	-	5.92	-	1.09	-	2.71	-	1.42	-
trans-carveol	1812	3.45	-	2.67	-	1.88	-	2.44	-	6.62	-	4.66	-	2.60	-	3.09	-	5.31	-	1.79	-	2.18	-	2.19	-
geraniol	1821	-	-	-	-	-	-	0.15	-	-	-	-	-	-	-	-	-	-	-	-	-	-	-	-	-
p-cymen-8-ol	1828	-	-	-	-	8.81	-	5.26	-	-	-	2.21	-	-	-	1.41	-	-	-	1.21	-	6.86	-	9.17	-
geranyl acetate	1835	-	-	-	-	0.28	-	0.56	-	-	-	-	-	0.09	-	-	-	-	-	-	-	0.88	-	-	-
cis-carveol	1845	-	-	3.15	-	-	-	5.97	-	-	-	-	-	-	-	6.93	-	-	-	-	-	-	-	6.31	-
trans-myrtanol	1854	-	-	-	-	2.18	-	1.19	-	-	-	-	-	-	-	-	-	-	-	-	-	0.98	-	1.17	-
cis-myrtanol	1855	-	-	0.60	-	-	-	-	-	-	-	-	-	-	-	-	-	-	-	-	-	-	-	-	-
p-cymen-9-ol	1878	-	-	-	-	0.80	-	0.70	-	-	-	-	-	-	-	0.71	-	-	-	-	-	-	-	0.91	-
piperitenone	1917	-	-	-	-	0.45	-	0.61	-	-	-	-	-	-	-	0.52	-	-	-	-	-	-	-	0.71	-
geranyl isovalerate	1957	1.50	1.08	1.12	0.82	0.15	0.91	1.48	0.72	0.72	0.23	0.09	-	0.65	1.17	1.44	-	0.18	0.13	0.09	0.12	0.28	0.33	0.24	0.30
3,6,6-trimethyl-2-norpinanol	1992	1.25	0.89	1.39	1.03	0.59	1.96	1.44	0.72	1.10	0.50	0.13	0.35	2.52	1.13	3.54	1.23	0.64	0.66	1.32	0.87	0.42	1.51	1.08	1.14
β-camphor	2038	-	-	-	-	-	-	0.53	-	-	-	-	-	-	-	0.75	-	-	-	-	-	-	-	-	-
p-cresol	2071	-	-	-	-	0.38	-	0.86	-	-	-	-	-	-	-	1.05	-	-	-	-	-	0.98	-	0.75	-
cuminyl alcohol	2090	-	-	-	-	0.63	-	0.71	-	-	-	-	-	-	-	0.82	-	-	-	-	-	-	-	1.09	-
pseudoionone	2126	-	-	-	-	0.46	-	0.39	-	-	-	-	-	-	-	0.46	-	-	-	-	-	-	-	0.29	-
longipinanol	2140	-	-	-	-	-	-	0.22	-	-	-	-	-	-	-	0.13	-	-	-	-	-	-	-	0.23	-
farnesol	2323	-	-	-	-	0.55	-	0.57	-	-	-	-	-	0.40	-	0.43	-	-	-	-	-	-	-	0.74	-
Total terpenes		110.43	20.33	119.56	2.33	141.07	21.44	151.51	7.32	579.01	1.9	49.02	0.35	281.13	2.82	114.87	1.23	107.32	1.76	22.72	2.36	87.34	3.0	184.51	2.1

The numbers in the sample codes indicate the fermentation temperature. G: glucose synthetic medium, S: Sucrose synthetic medium, M: maltose synthetic medium. I: Immobilized *Saccharomyces cerevisiae* AXAZ-1 cells on *Pistachia terebinthus* resin, F: Free cells of *Saccharomyces cerevisiae* AXAZ-1.
